# High-Resolution Optical Coherence Tomography Angiography Characteristics of Limbal Stem Cell Deficiency

**DOI:** 10.3390/diagnostics11061130

**Published:** 2021-06-21

**Authors:** Shobhit Varma, Swapna S. Shanbhag, Pragnya Rao Donthineni, Dilip Kumar Mishra, Vivek Singh, Sayan Basu

**Affiliations:** 1 The Cornea Institute, L V Prasad Eye Institute, Hyderabad 500034, Telangana, India; drshobhitvarma@gmail.com (S.V.); swapnashanbhag@lvpei.org (S.S.S.); drpragnyarao@lvpei.org (P.R.D.); 2 Ocular Pathology Services, L V Prasad Eye Institute, Hyderabad 500034, Telangana, India; dilipkumarmishra@lvpei.org; 3 Centre for Ocular Regeneration (CORE), L V Prasad Eye Institute, Hyderabad 500034, Telangana, India; viveksingh@lvpei.org; 4 Prof. Brien Holden Eye Research Centre (BHERC), L V Prasad Eye Institute, Hyderabad 500034, Telangana, India

**Keywords:** limbal stem cell deficiency, chemical burns, high resolution optical coherence tomography, high resolution optical coherence tomography angiography, ocular imaging

## Abstract

This study aimed to identify the anterior segment high-resolution optical coherence tomography (HR-OCT) and HR-OCT angiography (HR-OCTA) features suggestive of limbal stem cell deficiency (LSCD) as confirmed by both impression cytology (IC) and in vivo confocal microscopy (IVCM). This was a single-centre prospective cross-sectional study including 24 eyes of 22 patients with clinical suspicion of LSCD based on peripheral superficial corneal vascularisation and scarring. On IC and IVCM, performed and interpreted by blinded observers, 12 eyes each were diagnosed with and without LSCD. Additionally, 10 eyes of 5 healthy volunteers with no ocular pathology were also imaged. The 136 HR-OCT/A images of these 34 eyes were analysed with respect to 12 imaging parameters; the parameters most suggestive of LSCD were identified and the sensitivity and specificity were calculated. In the LSCD group, the most common aetiology was ocular chemical burns (83%), whereas in the non-LSCD group, the most common aetiology was viral keratitis (67%). Multiple logistic regression analysis revealed that mean epithelial reflectivity, mean stromal reflectivity, and mean superficial vascular density were the parameters that were diagnostic of LSCD on HR-OCT/A (*p* < 0.0001). A ratio of the mean epithelial reflectivity to stromal reflectivity of >1.29 corresponded with a high sensitivity (91.7%) and specificity (98.75%); while a mean superficial vascular density score of >0.38 corresponded with a sensitivity of 97.9% and specificity of 73.8%. In conclusion, HR-OCT/A as a non-invasive imaging modality could prove to be a useful tool for confirming the diagnosis of LSCD, with potential clinical and research applications.

## 1. Introduction

The cornea is an optically transparent structure and is responsible for three-fourths of the refractive power of the eye. Regularity of the surface epithelium is an important factor for maintaining this transparency. This continuous process of corneal epithelial cell renewal is normally sustained throughout life due to the presence of epithelial stem cells at the limbus [1]. The limbus is the annular transition zone between the transparent cornea and the opaque sclera and contains the palisades of Vogt, which houses the corneal epithelial stem cells [2,3]. Damage to the limbus secondary to injury or inflammation can lead to a state of corneal epithelial dysfunction, known as limbal stem cell deficiency (LSCD). The hallmark of LSCD is conjunctivalisation of the cornea. Since the barrier between the corneal and conjunctival epithelium is lost, the cornea is subsequently covered by conjunctival epithelium which is accompanied by corneal scarring and vascularisation. LSCD can thus cause a loss in corneal clarity, thus being a significant aetiology for corneal blindness. There are several aetiologies that can lead to LSCD such as ocular chemical burns, multiple ocular surgeries, Stevens–Johnson syndrome, mucous membrane pemphigoid, vernal keratoconjunctivitis, and aniridia [4].

The confirmation of the diagnosis of LSCD is the first critical step in managing this condition [5]. Historically, the most common diagnosis has been clinical [6], based on slit lamp examination where LSCD is characterised by absence of visible limbal palisades of Vogt, conjunctival overgrowth over the corneal surface with superficial corneal vascularisation and scarring and stippled fluorescein staining of the corneal surface. This method of interpretation is subjective with considerable interobserver variation and hence may not be considered very reliable. More objective methods that have been described to confirm the diagnosis of LSCD include corneal impression cytology (IC) [7] and in vivo confocal microscopy (IVCM) [8]. Corneal IC however is a contact procedure and has low sensitivity for diagnosing LSCD [9]. The IVCM is a three-dimensional real time imaging tool to assess LSCD, the results of which have high concordance with those of IC [10]. However, it is a semi-contact method, time-consuming, highly operator dependent, and provides a very small field of imaging. An objective confirmation of the diagnosis of LSCD, therefore is usually made with a combination of clinical examination with fluorescein staining, IC, and IVCM findings.

Over the last decade, high-resolution optical coherence tomography (HR-OCT) has emerged as a popular method of imaging for the anterior segment. The HR-OCT imaging of the cornea is a rapid, non-contact technique, with low operator dependence and provides instantaneous output and analysis with good histopathological correlation [11,12]. Previous studies have reported the usefulness of HR-OCT in cases of LSCD by assessing the limbal epithelial thickness [13], for visualising palisades [14], and quantifying the limbal stem cell niche [15]. Recently, the HR-OCT angiography (OCTA) has been used for the anterior segment for imaging corneal neovascularisation [16], limbal and peri-limbal vasculature [17]. However, features on HR-OCT and HR-OCT angiography that are sensitive and specific for LSCD, and could be used for diagnosis of LSCD have not yet been determined. Thus, in this paper, the authors have attempted to identify these features that could aid in rapid non-invasive and non-contact-based diagnosis of LSCD.

## 2. Materials and Methods

### 2.1. Study Design, Location, Duration and Participants

**Figure 1:**
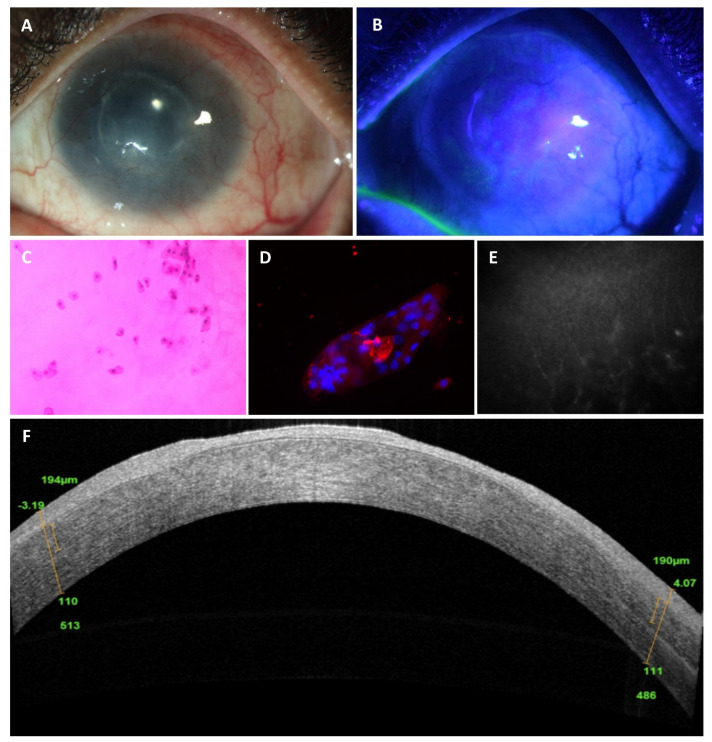
Characteristics of limbal stem cell deficiency (LSCD) clinically and with different imaging modalities. (**A**) Slit-lamp image of an eye with LSCD characterised clinically by loss of corneal clarity, corneal vascularisation, and scarring; (**B**) positive fluorescein staining; (**C**) impression cytology from the corneal surface showing Nelson Grade 3;* (**D**) presence of conjunctival cytokeratin (CK) marker (CK19) on immunohistochemistry; (**E**) decreased sub basal nerve plexus, altered basal cell layer morphology on in vivo confocal microscopy; (**F**) replacement of the dark hyporeflective corneal epithelial phenotype with bright and thick conjunctival phenotype on optical coherence tomography. * Nelson Grade 3—from ‘Nelson JD. Impression cytology. *Cornea* 1988, 7, 71–81.

This was a prospective cross-sectional imaging study done at the L.V. Prasad Eye Institute, Hyderabad, India between 1 August 2018 and 30 June 2019. All adult patients with peripheral corneal vascularisation and scarring with a clinical suspicion of LSCD in at least one eye were eligible to be included along with age and sex-matched healthy normal volunteers. All patients and volunteers underwent a comprehensive ocular examination including slit-lamp examination with fluorescein staining of the ocular surface. Next, all recruited patients underwent IC, IVCM, HR-OCT and HR- OCTA at baseline by single observers who were blinded to the clinical diagnosis during the initial sitting. Based on the findings noted on slit lamp photomicrographs, IC and IVCM, the patients were divided into 2 groups (i) LSCD and (ii) non-LSCD. Patients who refused to undergo all the required investigations, uncooperative patients, and patients in whom good quality imaging or sampling could not be performed were excluded from the study. Additionally, patients who underwent IC and IVCM but had discordance in the results between IC and IVCM and where LSCD or non-LSCD could not be confirmed, were excluded from this study. The HR-OCT/A images of the patients in the LSCD and non LSCD groups were then processed and analysed by observers who were blinded to the clinical diagnosis (one observer for each of the tests). Figure 1 shows the clinical appearance of an eye with LSCD and the characteristics on IC, IVCM, and HR-OCT.

### 2.2. Details on Imaging and Diagnostic Procedures

Slit-lamp photographs were captured before and after application of fluorescein-dye using cobalt blue light. Corneal IC was then performed, and the technique used has been described in detail previously [18]. Specimens then underwent hematoxylin and eosin staining and periodic acid Schiff staining to identify goblet cells. Analysis was done on the basis of Nelson’s grading system for impression cytology [19]. The specimens were further processed for immunohistochemistry, and the details for these techniques have been provided previously [7]. Antibodies against CK3 (cytokeratin), CK12, CK19, and MUC5AC were also tested. A diagnosis of LSCD was made for eyes which tested positive for CK 3, CK19, and MUC5AC.

IVCM was performed with the Confoscan 4 (Nidek Technologies Srl, Albignasego (PD), Italy). Confocal imaging of the central cornea, paracentral cornea and four locations of limbus was performed. Morphologic characteristics of the corneal epithelium and the nerves in the subbasal nerve plexus were studied. The limbus was examined for the presence of palisades of Vogt (poV). High-contrast digital images of all the corneal layers and limbus were acquired with a field of view of 300 × 300 mm^2^ and analysed using FIJI software [20]. Corneal images with altered epithelial cell morphology, sub-basal fibrosis, and reduction in nerve density in the sub-basal plexus, and limbal images with loss of poV on IVCM were diagnosed to have LSCD [8,10].

**Figure 2:**
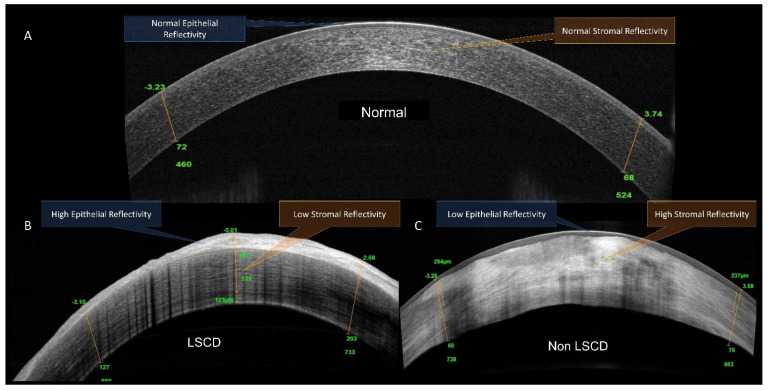
Corneal epithelial and stromal thickness and reflectivity on high resolution anterior coherence tomography (HROCT) line scan. (**A**) Normal eye; (**B**) eye with LSCD; (**C**) eye from the non-LSCD group.

**Figure 3:**
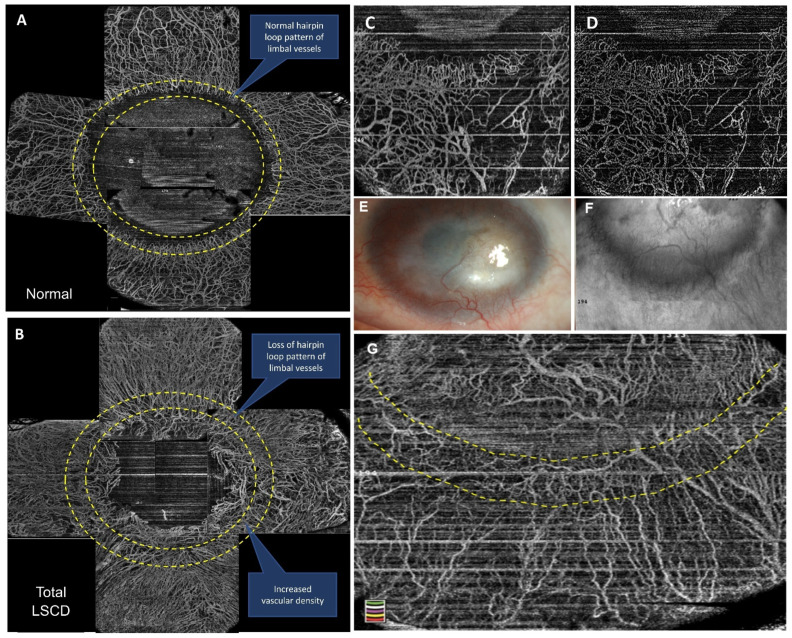
Calculation of vascular density on high-resolution optical coherence tomography (HR-OCT) angiography. (**A**) HROCT-A compilation of 360 degrees limbus in a normal eye; (**B**) HROCT-A compilation of 360 degrees limbus in an eye with total LSCD; (**C**) original image before Image thresholding; (**D**) image after image thresholding; (**E**) eye with LSCD; (**F**) HROCT-A infra-red image of the inferior half of the same eye; (**G**) determining the region of interest (ROI)—manual demarcation of inner limbus and automated selection of ROI.

HR-OCT was performed in all eyes on the central and paracentral cornea (Optovue Inc, Fremont, CA, USA). The cross-line scan mode was used to obtain the images of the central cornea. HR-OCTA was performed by a technician using the AngioVue OCTA system (Optovue, Inc., Fremont, CA, USA) in all four limbal quadrants (superior, inferior, nasal and temporal) with adjoining bulbar conjunctiva. The technique of HR-OCTA imaging that the authors performed has been explained by the authors previously [17]. Twelve parameters that were analysed for each quadrant on OCT and OCTA imaging were: (i) epithelial thickness (Figure 2), (ii) stromal thickness, (iii) ratio of epithelial thickness to stromal thickness, (iv) mean and mode of epithelial reflectivity (Supplementary file), (v) mean and mode of stromal reflectivity (Supplementary file), (vi) ratio of mean and mode of stromal reflectivity, (vii) mean total limbal vascular density, (viii) mean superficial limbal vascular density (Figure 3), (ix) mean total vascular density into bin distribution, (x) mean superficial vascular density into bin distribution, (xi) ratio of segmented to total vascular density, and (xii) maximum peak bin distribution frequency in total and segmented vascular density.

### 2.3. Image Analysis for HR-OCTA Images

Images were analysed in en-face custom mode by two settings: total vascular density and superficial vascular density. Further details of image analysis for these two settings are mentioned in Supplementary file The post-processing of OCTA images was performed on the Spyder version 3.3.3 [21]. An algorithm was custom written on this open-source cross platform integrated development environment for scientific programming where Python language was used. The primary purpose of the algorithm design was to generate an imaging biomarker that could quantitatively capture the visual observations of changes in vascular density distribution in the human eye as a function of radial and angular distances from the centre of the eye. This was used to generate the vascular density at limbus area for LSCD and non-LSCD cases. Steps of image processing are mentioned in Supplementary file.

### 2.4. Statistical Analysis

MedCalc (version 19.0.4, MedCalc Software, Mariakerke, Belgium) statistical software was used for data analysis. A two-tailed P value less than 0.05 was considered statistically significant. Descriptive statistics using mean ± standard deviation, median ± inter-quartile range (IQR) and mode were used to elucidate the demographic data. Multiple logistic regression analysis using Akaike’s information criteria (AIC) with step wise elimination modelling was used to identify the HR-OCT angiography parameters significantly associated with the confirmed diagnosis of LSCD. To ensure that the study was adequately powered for multiple regression analysis, the sample size was calculated as a minimum of 20 eyes with 10 eyes in each group (LSCD and non-LSCD). Subsequently, a Receiver Operating Characteristic (ROC) curve analysis was performed to detect the threshold value for optimal sensitivity and specificity (using criterion corresponding with highest Youden index) of diagnosis of LSCD for each parameter identified by multiple logistic regression modelling. Positive and negative predictive values for the threshold scores were also calculated along with 95% confidence intervals.

## 3. Results

### 3.1. Demographics and Baseline Data

This study included 24 eyes of 22 patients with clinical suspicion of LSCD. On IC and IVCM, 12 eyes of 11 patients were diagnosed to have LSCD (both IC and IVCM positive for LSCD) while 12 eyes of 11 patients were diagnosed to not have LSCD (non-LSCD group; both IC and IVCM negative). Additionally, ten eyes of five volunteers were imaged that had no ocular pathology (normal appearing limbus with no history of contact lens wear). Among the LSCD group, there were nine males (82%) and two females (18%). The mean age of patients in this group was 26.8 ± 8.7 years. The most common aetiology for LSCD was ocular chemical burns (10/12 eyes: 83%) with BCVA ranging from 20/30 to hand movement. In the non-LSCD group, there were eight males (73%) and three females (27%). The mean age of patients in this group was 38.45 ± 15.6 years. The most common aetiology was corneal vascularisation and scarring secondary to viral keratitis (8/12; 67%) with BCVA ranging from 20/20 to counting fingers at 1 metre. Out of five normal subjects, three (60%) were male while two (40%) were female. The mean age of patients in this group was 27.6 years ± 2.9 years. A total of 136 quadrants of 34 eyes were analysed by HR-OCTA imaging, out of which 48 quadrants (35%) had confirmed LSCD while 88 quadrants (65%) belonged to non-LSCD eyes.

### 3.2. HR-OCT Imaging Parameters Diagnostic of LSCD

**Figure 4:**
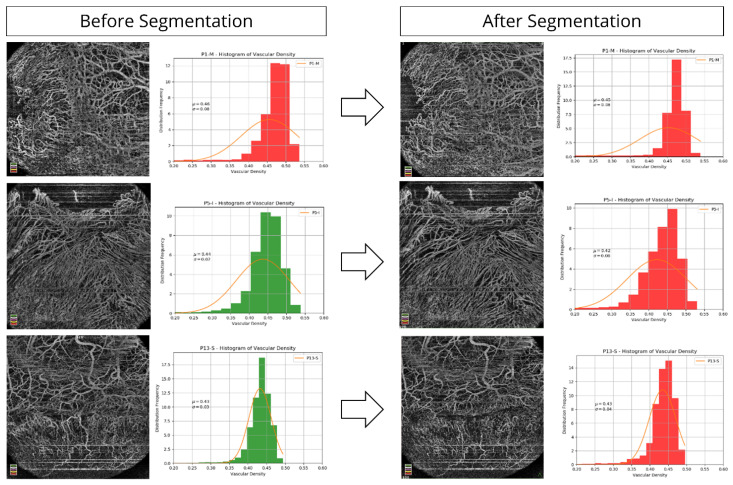
Vascular density analysis after segmentation at the limbus on HR-OCT angiography in eyes with LSCD.

**Figure 5:**
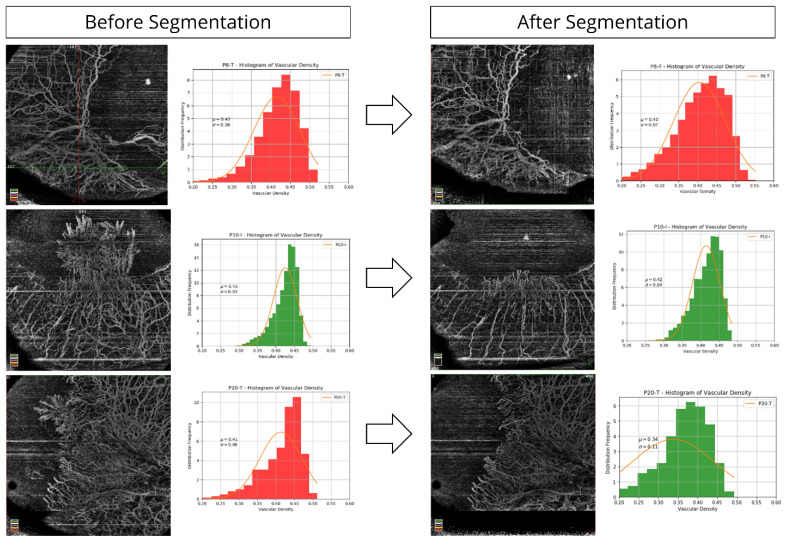
Vascular density analysis after segmentation at the limbus on HR-OCT angiography in eyes without LSCD.

**Figure 6:**
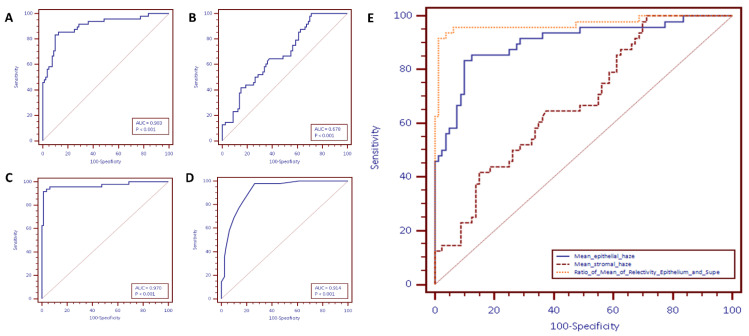
Receiver operating characteristic (ROC) curves for all the parameters that were significant for differentiating between eyes with LSCD and non-LSCD on high-resolution optical coherence tomography (HROCT) and HROCT angiography. (**A**) ROC curve for mean epithelial reflectivity; (**B**) ROC curve for mean stromal reflectivity; (**C**) ROC curve for ratio of mean epithelial to stromal reflectivity; (**D**) ROC curve for mean superficial vascular density; (**E**) combined ROC curves for mean, epithelial reflectivity, mean stromal reflectivity, and ratio of mean epithelial to stromal reflectivity.

Multiple logistic regression analysis of the 12 imaging parameters, revealed three parameters to be significantly different in eyes with LSCD as compared to the non-LSCD group and normal eyes (data provided in Supplementary file). These statistically significant parameters were mean epithelial reflectivity, mean stromal reflectivity, and mean superficial vascular density. The first two parameters require line scans on HR-OCT, while the third parameter requires HR-OCTA. Mean epithelial reflectivity had a coefficient of 0.12156 (SE 0.031), mean stromal reflectivity had a coefficient of −0.08 (SE 0.02) and mean superficial vascular density had a coefficient of 51.27 (SE 17.44). The calculation of the total vascular density and the superficial vascular density in eyes with LSCD and in eyes with Non-LSCD on HR-OCT angiography is shown in Figure 4 and Figure 5. Area under the ROC curve (Supplementary file) for logistic regression for the above three parameters is 0.992 with standard error of 0.007 and 95% confidence interval of 0.957 to 1.00. For the three criteria found to be statistically significant (*p* < 0.0001), more details about individual parameters are mentioned in Supplementary file. (mean epithelial reflectivity; mean stromal reflectivity; ratio of mean epithelial reflectivity to stromal reflectivity; mean superficial vascular density). Figure 6 shows the ROC curves of all the three parameters.

### 3.3. Sensitivity, Specificity, Positive and Negative Predictive Values of HR-OCT Angiography Parameters of LSCD

Mean epithelial reflectivity (Figure 6A): A score of >142.9 corresponded with the highest Youden index. The sensitivity at this threshold was 83.33 (95% CI: 69.8 to 92.5) and specificity of 90.00 (81.2 to 95.6). The positive likelihood ratio was 8.33 (95% CI = 4.3 to 16.3), while the negative likelihood ratio was 0.19 (95% CI = 0.10 to 0.4).

Mean stromal reflectivity (Figure 6B): A score of ≤151.8 corresponded with the highest Youden index. The sensitivity at this threshold was 100 (95% CI: 92.6 to 100) and specificity of 28.75 (19.2 to 40). The positive likelihood ratio was 1.40 (95% CI = 1.2 to 1.6), while the negative likelihood ratio was 0.

Ratio of mean epithelial reflectivity to stromal reflectivity (Figure 6C): A score of >1.29 corresponded with the highest Youden index. The sensitivity at this threshold was 91.67 (95% CI: 80 to 97.7) and with specificity of 98.75 (93.2 to 100). The positive likelihood ratio was 73.3 (95% CI = 10.4 to 515.2), while the negative likelihood ratio was 0.084 (95% CI = 0.02 to 0.2).

Mean superficial vascular density (Figure 6D): A score of >0.38 corresponded with the highest Youden index. The sensitivity at this threshold was 97.9 (95% CI: 88.9 to 99.9) and specificity of 73.75 (62.7 to 83). The positive likelihood ratio was 3.73 (95% CI = 2.6 to 5.4), while the negative likelihood ratio was 0.028 (95% CI = 0.02 to 0.2). 

## 4. Discussion

This study aimed to identify the HR-OCT/A features that are most reliable and accurate for the diagnosis of LSCD, as confirmed by a combination of IC and IVCM. The findings of this study suggest that three HR-OCT/A parameters (epithelial reflectivity, stromal reflectivity and limbal superficial vascular density) were highly predictive of the diagnosis of LSCD. The field of limbal biology, limbal imaging and limbal stem cell transplantation for the treatment of LSCD is rapidly evolving [22]. HR-OCT is a popular imaging modality commonly used by cornea specialists, however the technology of non-invasive angiography of the anterior segment using HR-OCT is a relatively recent advancement. Since HR-OCT angiography is a non-contact, non-invasive, real time, and high-resolution imaging modality with low operator dependence, it can potentially be useful both for the diagnosis and treatment of LSCD. Importantly, the epithelial to stromal reflectivity ratio [23], which has high sensitivity as well as specificity, is based on line-scans that can be obtained using anterior segment HR-OCT platforms that do not include angiography modules.

The current gold standards for LSCD diagnosis are IC and IVCM, however they are not routinely available in a cornea clinic. IC requires specialised techniques for staining and requires trained staff and a fully equipped laboratory to process and evaluate the specimens. The evaluation is time-consuming, thus delaying the diagnosis. Additionally, the probability of false positive or false negative results is high due to tissue contamination while obtaining or processing the samples. IVCM is an expensive machine, not commonly available in most cornea clinics, highly operator dependent, and obtaining good quality images in eyes with LSCD may be a significant challenge in eyes with significant corneal scarring or conjunctivalisation. Being a semi-contact method of imaging, it requires patient cooperation in image acquisition. Thus, to overcome these limitations most cornea specialists rely on clinical slit lamp examination for diagnosis. Our study was unique because the preliminary diagnosis of LSCD was not made on clinical examination alone, but IC and IVCM were considered as gold standard diagnostic tools for diagnosis of LSCD.

Out of the three parameters of HR-OCT angiography discussed above, epithelial and stromal reflectivity can be detected easily without any need for complex data processing. These values can also be obtained using HR-OCT machines that are not necessarily equipped to perform angiography. Mean epithelial reflectivity is a better parameter than stromal reflectivity. Furthermore, when the ratio of these two parameters is taken into consideration, a steep improvement is observed in the sensitivity and specificity of the test (Figure 6E). Conjunctivalisation of cornea leads to a change in the epithelium signal interference pattern resulting in an extremely high reflective signal intensity on OCT. The demarcation between the epithelium and the stroma is sharp due to dysplastic changes in the epithelium. The stromal reflectivity is normal in cases of LSCD with mild to moderate epithelial thickness. However, in highly reflective epithelium and cases with significantly higher epithelial thickness, the stromal reflectivity might be lower than normal due to back shadowing of the epithelium. Thus, the ratio of the two parameters amplifies the sensitivity and specificity more than either parameter in isolation.

In a non-LSCD eye with peripheral corneal vascularisation and scarring, the epithelium is unaffected and has normal epithelial reflectivity on OCT. However, there is contrasting increase in signal intensity of the stroma due to scarring. We found that measuring reflectivity can be an efficient way to differentiate eyes with LSCD from other aetiologies which may seem similar to LSCD. Previously, Karp et al. utilised epithelial hyperreflectivity on OCT to diagnose cases of ocular surface squamous neoplasia [24]. Additionally, Liang et al. described lower values of central corneal epithelial thickness and maximum limbal epithelial thickness on OCT as reliable parameters for confirmation of the diagnosis of LSCD [13]. However, epithelial thickness parameters depend on stage and chronicity of LSCD, hence could be useful for quantitatively staging LSCD but may not be a reliable criterion for diagnosis of LSCD. Binotti et al. described the use of OCT angiography for assessing eyes with LSCD by analysing changes in vascular parameters such as the maximum corneal vascular extension from the limbus to the furthest vessel over the cornea, and corneal vascular thickness from the most superficial to the deepest corneal vessel [25]. However, in the study by Binotti et al., the aetiology for LSCD was LSCD secondary to contact lens wear, herpetic keratitis, or chronic allergic keratitis, in contrast to our study where the aetiology for a majority of cases was LSCD secondary to ocular chemical injuries. In our study, eyes with herpetic keratitis did not show features of LSCD on IC or IVCM. Additionally, studies by Liang et al. and Binotti et al. compared these characteristics in eyes with LSCD with eyes of healthy volunteers, without comparing them to cases which were not LSCD, but were clinically suspicious of the same. Additionally, corneal neovascularisation is not necessarily a hallmark of LSCD, and could also occur in eyes without the aetiology being LSCD. Hence, analysing only vascular parameters could lead to erroneous conclusions.

Conjunctivalisation of the cornea is a hallmark of LSCD. The blood vessels within the fibrovascular pannus that grows over the cornea in eyes with LSCD are derived from the superficial episcleral vascular plexus, which also supplies the conjunctiva. These pathological vessels are superficial in location and can be differentiated from deeper vascularisation of the peripheral cornea, which is unrelated to LSCD. When HR-OCT angiographic images are segmented for the superficial layers, there is minimal change in vascular density in cases of true LSCD (Figure 4). However, in eyes that do not have LSCD but have deeper vascularisation, due to other causes of corneal inflammation such as herpetic keratitis, superficial segmentation results in significant reduction in the limbal vascular density and therefore the differences between LSCD and non-LSCD eyes become easily discernible (Figure 5). Angiography images need complex technologies for analysis; however, they aid in validating the diagnostic sensitivity and specificity significantly and hence could prove to be a benchmark for analysis in the future.

The strengths of our study include the fact that we compared the HR-OCT and HR-OCTA parameters in eyes with LSCD to eyes with LSCD mimickers, and in eyes of healthy volunteers. Additionally, our study included more typical causes of LSCD such as ocular chemical burns. The main limitations of our study include lack of clinical staging of LSCD, and relatively modest number of eyes included per group. Some inherent limitations also exist with the OCTA imaging technology for the anterior segment, such as longer image acquisition time as compared to posterior segment and need for good co-operation from the patient. No clock hour demarcation settings exist in the software for accurate 360 degree scanning of limbus and the cross-sectional limbal images taken at the site of vascular imaging have a low resolution. Additionally, as the in-built software is custom built for retina, the settings need to be modified before imaging and image analysis. Currently it takes about 5 min for the reflectivity measurement and another 5–10 min for the vascular density measurement. Hopefully with further advances in HR-OCT platforms there will be automated settings for the anterior segment angiography scans and integrated software for reflectivity and vascular density assessment, which will significantly reduce the post-processing time and improve the applicability of this modality. Previous studies have shown good repeatability and reproducibility for measuring parameters such as central corneal thickness using HR-OCT [26]. However, as we aimed to explore the OCT characteristics of LSCD in this study, we did not look at the intra and interobserver variability. It would be interesting to look at the repeatability of the measurements such as reflectivity and vascular density using HR-OCT in future studies.

## 5. Conclusions

In conclusion, this study aimed to identify the HR-OCT angiography features that are most reliably diagnostic of LSCD as compared to the current gold-standards of a combination of clinical examination, IC, and IVCM. Since HR-OCT angiography is a non-contact, non-invasive, real time, and high-resolution imaging modality with low operator dependence, it can potentially be very useful both for the objective diagnosis of LSCD as well as for monitoring the success of different stem cell-based therapies in clinical trials.

## Data Availability

All the data relevant to study has been provided in the manuscript and the supplementary file.

## Supplementary Materials

The supplementary file describes the image processing, analysis and the statistical analysis of the data on various parameters studied in this paper. The following are available online at https://www.mdpi.com/article/10.3390/diagnostics11061130/s1.
