# Supplementary material for: High-Resolution Optical Coherence Tomography Angiography Characteristics of Limbal Stem Cell Deficiency
Source: Diagnostics (Basel). 2021 Jun 21;11(6):1130. doi: 10.3390/diagnostics11061130 (PMC8233779; doi:10.3390/diagnostics11061130)
Supplement: Supplementary file 1 [file diagnostics-11-01130-s001.zip › diagnostics-1240199-supplementary.pdf]

Supplementary File

1. **HR-OCT Reflectivity Analysis**

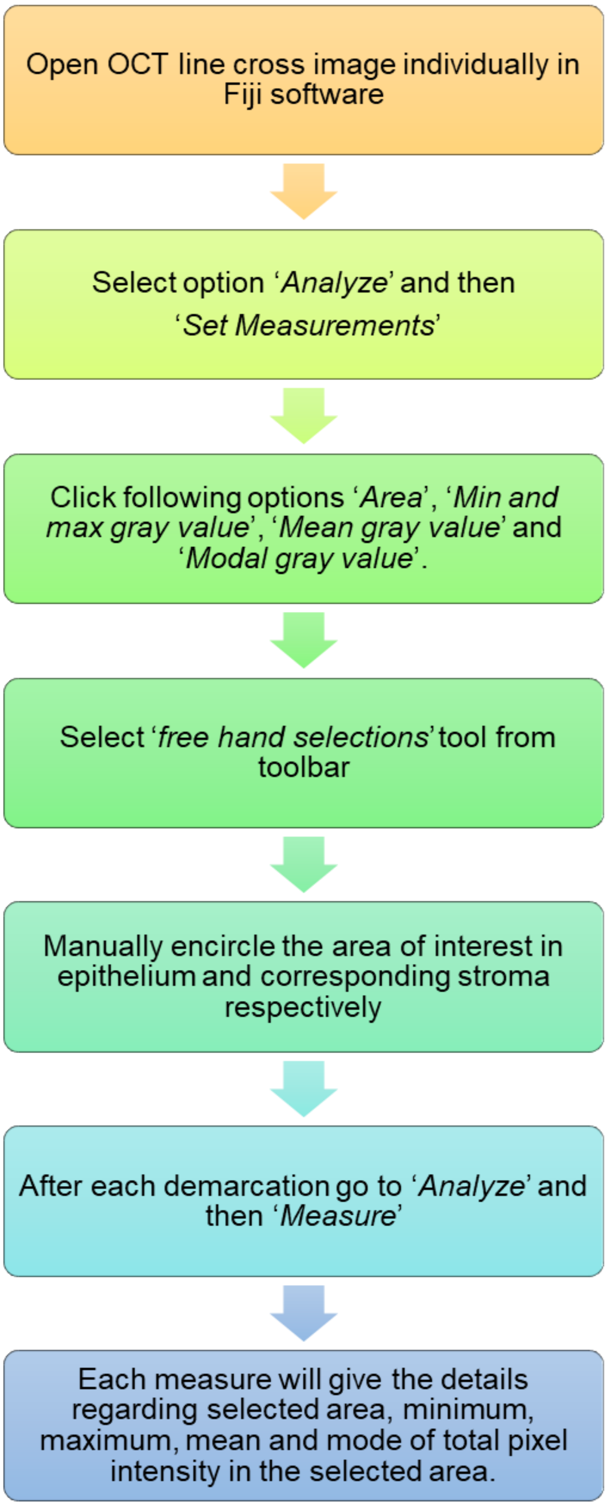

'Set Measurements' option box in Fiji

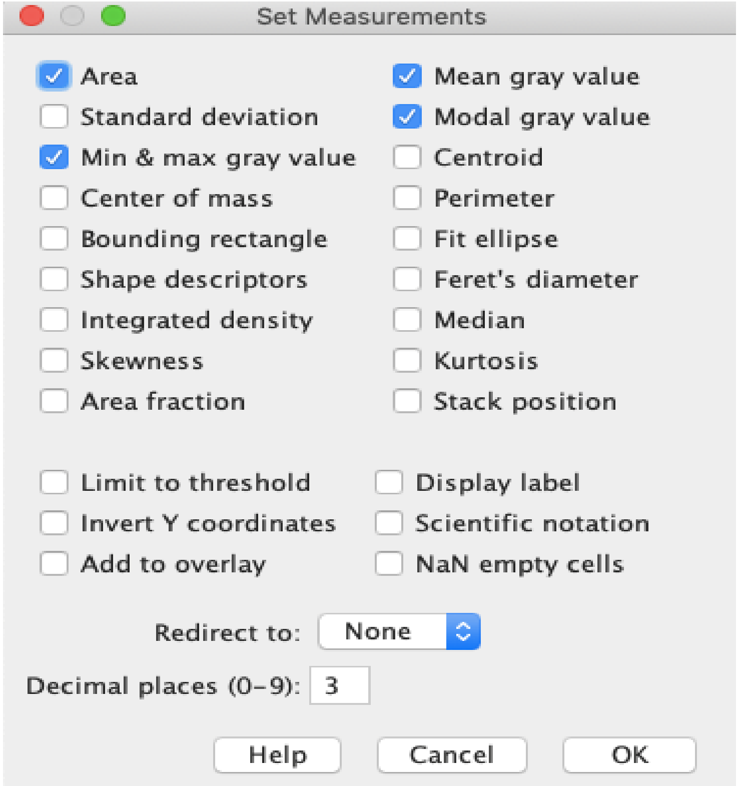

2 Epithelial and stromal reflectivity analysis:

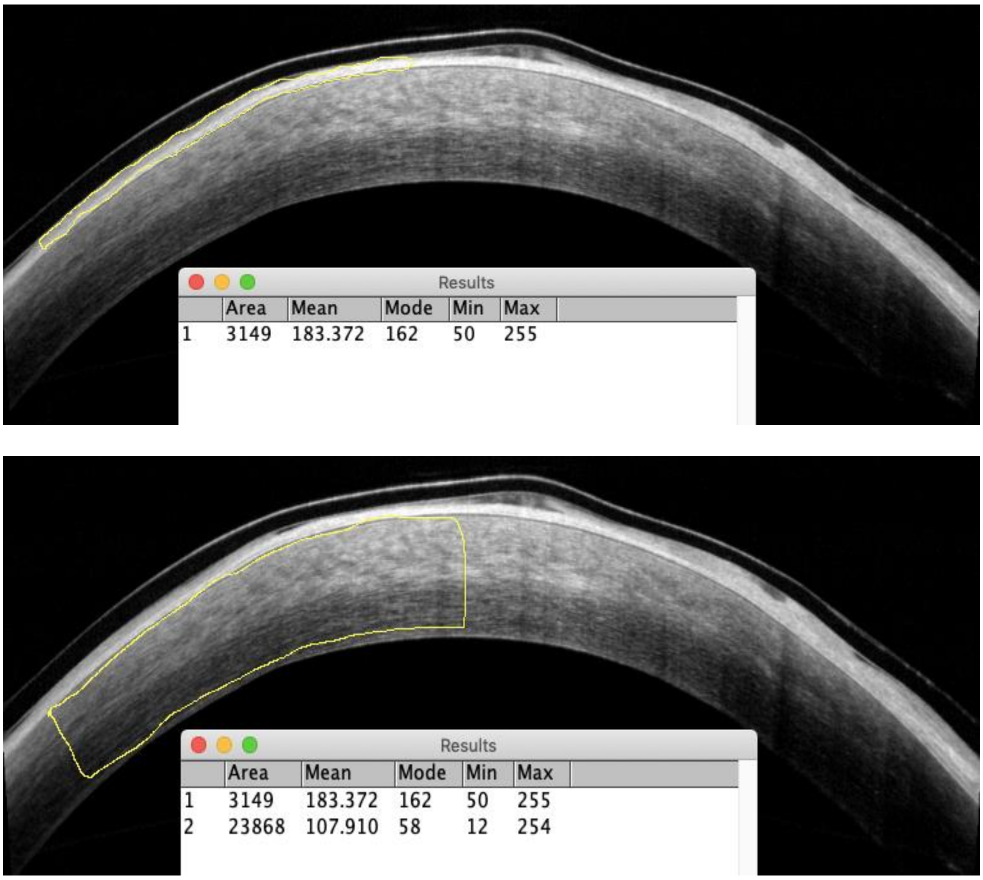

Supplementary figure S1 : *Top* – Epithelial reflectivity analysis,

Bottom – Stromal reflectivity analysis

Upper RPE offset is set at -800. On setting the lower RPE offset at 1200 the corresponding scanning zone at limbus covers the full thickness of sclera which is indicated in the cross-sectional side image. (Supplementary Fig S2)

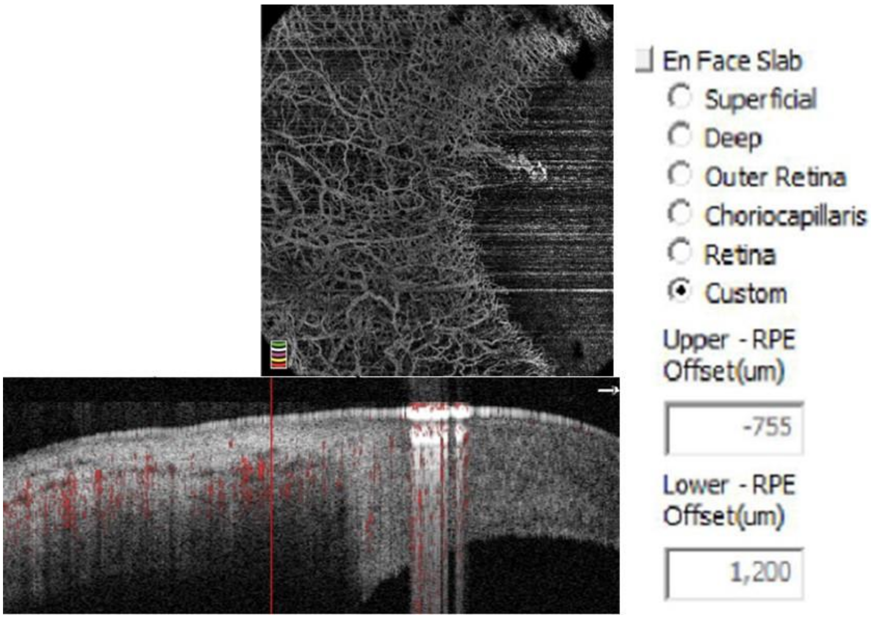

Supplementary Figure S2. Settings for the parameter – Total vascular density (total limbal vasculature)

B) <u>Superficial vascular density:</u>

Similar to description above the upper RPE offset is set at -800. While the lower RPE is set at 243. At this setting the scan zone covers scleral thickness till superficial stroma of cornea thus giving the limbal vasculature till the level of superficial limbus. (Supplementary Fig S3)

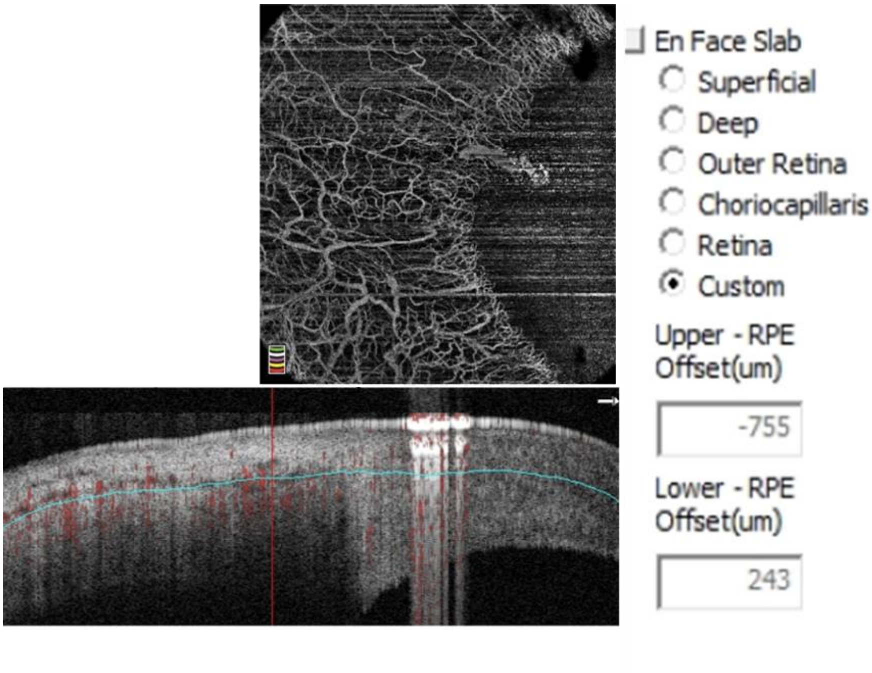

Supplementary Figure S3. Scan settings for superficial limbal vasculature

3. **Post-Processing of HR-OCT Angiography Images**

The first image processing step involved identification and isolation of vasculature from the background tissue. This was accomplished by a simple operation of thresholding using a noise floor of ~14% of full signal range (Supplementary Fig S4). To ensure that density is computed at every region only in the peripheral zone outward from the eye centre at the beginning of the vascular margin, a region of interest was demarcated isolating this region.

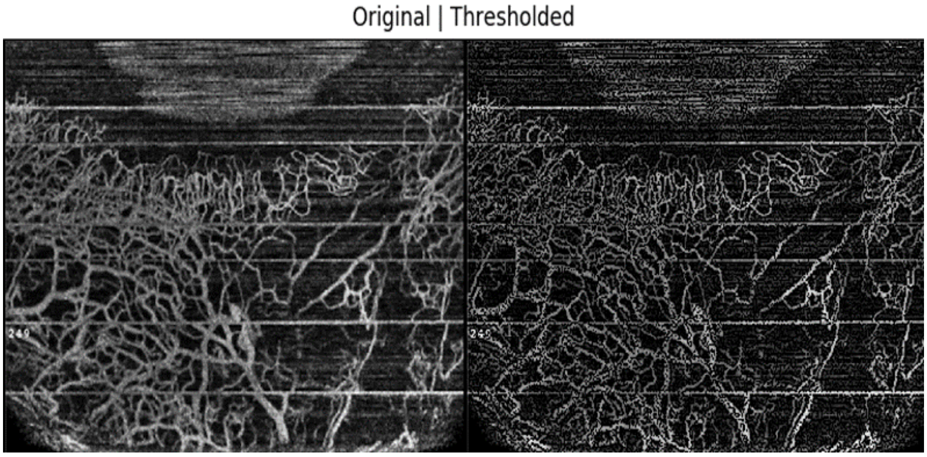

Supplementary Figure S4. Image thresholding for analysis

On every quadrant of the Image, the inner arc of the limbus area was marked which is an input to the software. The inner arc was determined manually for each image as automated arc recognition was not possible in LSCD cases with marked distortion of limbal vasculature.

Three points along the limbus arc were determined. X and Y coordinates of each point were noted and with the help of a custom based three points calculator, the final arc coordinates of the arc were calculated. This was entered in the program before each image processing. The arc coordinates were same for analysis of superficial and total vascular density of a same quadrant of a particular patient.

The region containing the vasculature was then used to compute a vascular density map. The eye is divided into small tiles of 3 x 3 pixels and each tile is visited one-by-one. A region-of-interest (ROI) is selected around the centre of a tile by extracting a small angular sector of ± 3 degrees and radial distance of ± 20 pixels. This region-of-interest has an area say 'A' pixels.

The total vascular pixels within this ROI are then counted as those pixels where the signal value is greater than a minimum threshold corresponding to a noise floor of ~14% (intensity value=36, over a full range value of 255).

Let's say this count is 'V' pixels. The ratio of V/A is defined as vascular density (VD).

$$Vascular\ Density\ (VD) = \frac{V}{A} = \frac{\sum (Vasculature\ pixels\ in\ ROI)}{\sum (All\ pixels\ in\ ROI)}$$

This is a normalized value bounded between zero and one.

This value is computed at all points in the image and stored as a vascular density map (Supplementary Fig S5). After the generation of the Vascular Density Map (VDM), a histogram is computed to generate summary description statistics of vascular density distribution across the whole eye.

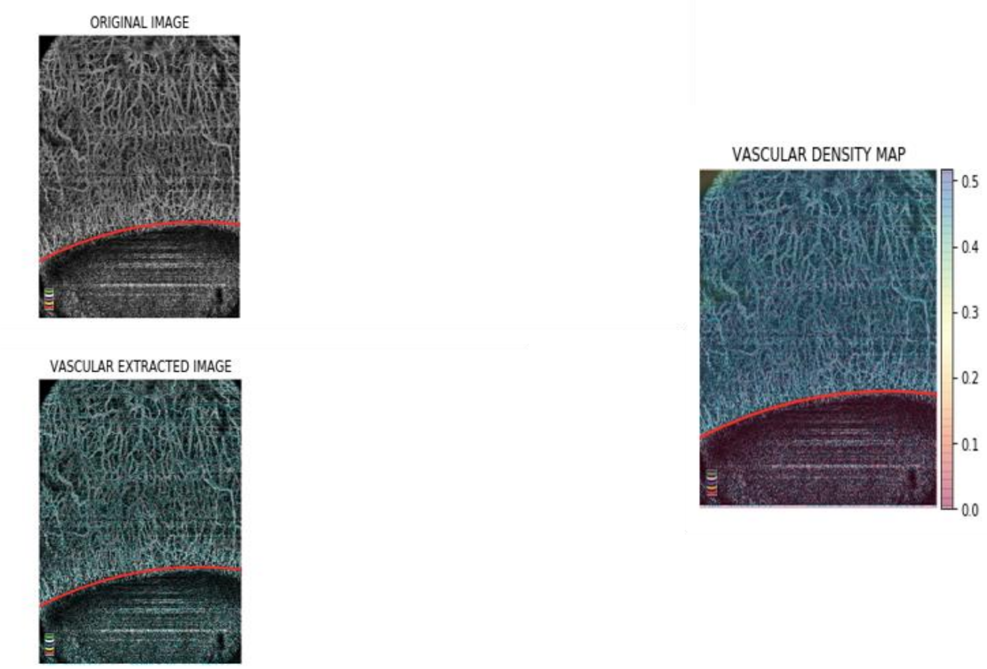

Supplementary Figure S5. Colour coded vascular density map during analysis

Tables

Table S1: Overall Model Fit

**Null model -2 Log Likelihood**	169.360
**Full model -2 Log Likelihood**	23.927
**Chi-squared**	145.433
**DF**	3
**Significance level**	P < 0.0001
**Cox and Snell R^2**	0.6790
**Nagelkerke R^2**	0.9254

Table S2: Coefficients and Standard Errors

Variable	Coefficient	Std. Error	Wald	P
Mean epithelial reflectivity	0.12156	0.031020	15.3572	0.0001
Mean stromal reflectivity	-0.083103	0.027061	9.4310	0.0021
Mean Superficial Vascular density	51.27159	17.44725	8.6357	0.0033
Constant	-28.14779	8.37562	11.2942	0.0008

Table S3: Hosmer and Lemeshow test

**Chi-squared**	4.8937
**DF**	8
**Significance level**	P = 0.7689

Table S4: Contingency table for Hosmer and Lemeshow test

Group	Y=0		Y=1		Total
	Observed	Expected	Observed	Expected	
1	13	12.999	0	0.000669	13
2	13	12.994	0	0.00558	13
3	13	12.980	0	0.0201	13
4	13	12.938	0	0.0622	13
5	12	12.789	1	0.211	13
6	13	11.643	0	1.357	13
7	3	3.461	10	9.539	13
8	0	0.165	13	12.835	13
9	0	0.0295	13	12.970	13
10	0	0.00136	11	10.999	11

Table S5: Classification table (cut-off value p=0.5) Intervals

Actual group	Predicted group		Percent correct
	0	1	
Y = 0	78	2	97.50%
Y = 1	3	45	93.75%
Percent of cases correctly classified			96.09%

Table S6: ROC curve analysis

**Area under the ROC curve (AUC)**	0.992
**Standard Error**	0.00701
**95% Confidence interval**	0.957 to 1.000

First parameter found to be significant - Mean epithelial reflectivity

On ROC analysis, the area under the curve (AUC) was 0.903 (95% CI=0.83 to 0.94) with a z statistic of 13.54 (p<0.0001). A score of >142.862 corresponded with the highest Youden index. The sensitivity at this threshold was 83.33 (95% CI: 69.8 to 92.5) and specificity of 90.00 (81.2 to 95.6). The positive likelihood ratio was 8.33 (95% CI=4.3 to 16.3), meaning a score of >142.862 was 8.33 times more likely in eyes with LSCD, than in eyes with non-LSCD. The negative likelihood ratio was 0.19 (95% CI=0.10 to 0.4), meaning a score of ≤142.862 was 19 times more likely in eyes with non-LSCD than in eyes with LSCD.

Table S7: Area under ROC curve for mean epithelial reflectivity

**Area under the ROC curve (AUC)**	0.903
**Standard Error [a]**	0.0297
**95% Confidence interval [b]**	0.838 to 0.948
**z statistic**	13.541
**Significance level P (Area=0.5)**	<0.0001

[a] DeLong et al., 1988
[b] Binomial exact

Table S8: Youden index for mean epithelial reflectivity

**Youden index J**	0.7333
**Associated criterion**	>142.862
**Sensitivity**	83.33
**Specificity**	90.00

Table S9: Criterion values and coordinates of the ROC curve

Criterion	Sensitivity	95% CI	Specificity	95% CI	+LR	95% CI	-LR	95% CI
>130	91.67	80.0 - 97.7	71.25	60.0 - 80.8	3.19	2.2 - 4.5	0.12	0.05 - 0.3
>130.798	89.58	77.3 - 96.5	71.25	60.0 - 80.8	3.12	2.2 - 4.5	0.15	0.06 - 0.3
>130.975	89.58	77.3 - 96.5	72.50	61.4 - 81.9	3.26	2.3 - 4.7	0.14	0.06 - 0.3
>131.027	87.50	74.8 - 95.3	72.50	61.4 - 81.9	3.18	2.2 - 4.6	0.17	0.08 - 0.4
>132.456	87.50	74.8 - 95.3	75.00	64.1 - 84.0	3.50	2.4 - 5.2	0.17	0.08 - 0.4

>132.638	85.42	72.2 - 93.9	75.00	64.1 - 84.0	3.42	2.3 - 5.1	0.19	0.10 - 0.4
>140.032	85.42	72.2 - 93.9	87.50	78.2 - 93.8	6.83	3.8 - 12.3	0.17	0.08 - 0.3
>141.93	83.33	69.8 - 92.5	87.50	78.2 - 93.8	6.67	3.7 - 12.1	0.19	0.1 - 0.4
**>142.862**	**83.33**	**69.8 - 92.5**	**90.00**	**81.2 - 95.6**	**8.33**	**4.3 - 16.3**	**0.19**	**0.10 - 0.4**
>152.93	70.83	55.9 - 83.0	90.00	81.2 - 95.6	7.08	3.6 - 14.0	0.32	0.2 - 0.5
>152.991	70.83	55.9 - 83.0	91.25	82.8 - 96.4	8.10	3.9 - 16.8	0.32	0.2 - 0.5
>153.78	66.67	51.6 - 79.6	91.25	82.8 - 96.4	7.62	3.7 - 15.9	0.37	0.2 - 0.5
>153.891	66.67	51.6 - 79.6	92.50	84.4 - 97.2	8.89	4.0 - 19.7	0.36	0.2 - 0.5

Second parameter found to be significant – Mean stromal reflectivity

On ROC analysis, the area under the curve (AUC) was 0.678 (95% CI=0.590 to 0.758) with a z statistic of 3.741 (p = 0.0002). A score of ≤151.8 corresponded with the highest Youden index. The sensitivity at this threshold was 100 (95% CI: 92.6 to 100) and specificity of 28.75 (19.2 to 40). The positive likelihood ratio was 1.40 (95% CI=1.2 to 1.6), meaning a score of ≤151.8 was 1.4 times more likely in eyes with LSCD, than in eyes with non-LSCD. The negative likelihood ratio was 0.

Table S10: Area under ROC curve for mean stromal reflectivity

**Area under the ROC curve (AUC)**	0.678
**Standard Error** [a]	0.0476
**95% Confidence interval** [b]	0.590 to 0.758
**z statistic**	3.741
**Significance level P (Area=0.5)**	0.0002

[a] DeLong et al., 1988. [b] Binomial exact

Table S11: Youden index for mean stromal reflectivity

**Youden index J**	0.2875
**Associated criterion**	≤151.8
**Sensitivity**	100.00
**Specificity**	28.75

Table S12: Criterion values and coordinates of the ROC curve

Criterion	Sensitivity	95% CI	Specificity	95% CI	+LR	95% CI	-LR	95% CI
≤120.555	75.00	60.4 - 86.4	43.75	32.7 - 55.3	1.33	1.0 - 1.7	0.57	0.3 - 1.0
≤123.89	75.00	60.4 - 86.4	41.25	30.4 - 52.8	1.28	1.0 - 1.6	0.61	0.3 - 1.1
≤124.8	79.17	65.0 - 89.5	41.25	30.4 - 52.8	1.35	1.1 - 1.7	0.51	0.3 - 0.9
≤126.242	79.17	65.0 - 89.5	38.75	28.1 - 50.3	1.29	1.0 - 1.6	0.54	0.3 - 1.0
≤129.591	85.42	72.2 - 93.9	38.75	28.1 - 50.3	1.39	1.1 - 1.7	0.38	0.2 - 0.8
≤130.743	85.42	72.2 - 93.9	37.50	26.9 - 49.0	1.37	1.1 - 1.7	0.39	0.2 - 0.8

≤133.8	87.50	74.8 - 95.3	37.50	26.9 - 49.0	1.40	1.1 - 1.7	0.33	0.1 - 0.7
≤134.966	87.50	74.8 - 95.3	33.75	23.6 - 45.2	1.32	1.1 - 1.6	0.37	0.2 - 0.8
≤135.133	89.58	77.3 - 96.5	33.75	23.6 - 45.2	1.35	1.1 - 1.6	0.31	0.1 - 0.7
≤135.442	89.58	77.3 - 96.5	32.50	22.4 - 43.9	1.33	1.1 - 1.6	0.32	0.1 - 0.8
≤135.721	91.67	80.0 - 97.7	32.50	22.4 - 43.9	1.36	1.1 - 1.6	0.26	0.10 - 0.7
≤137.653	91.67	80.0 - 97.7	31.25	21.3 - 42.6	1.33	1.1 - 1.6	0.27	0.10 - 0.7
≤137.714	93.75	82.8 - 98.7	31.25	21.3 - 42.6	1.36	1.2 - 1.6	0.20	0.06 - 0.6
≤138.154	93.75	82.8 - 98.7	30.00	20.3 - 41.3	1.34	1.1 - 1.6	0.21	0.07 - 0.7
≤144.169	97.92	88.9 - 99.9	30.00	20.3 - 41.3	1.40	1.2 - 1.6	0.069	0.010 - 0.5
≤149.201	97.92	88.9 - 99.9	28.75	19.2 - 40.0	1.37	1.2 - 1.6	0.072	0.01 - 0.5
**≤151.8**	**100.00**	**92.6 - 100.0**	**28.75**	**19.2 - 40.0**	**1.40**	**1.2 - 1.6**	**0.00**	
≤232.513	100.00	92.6 - 100.0	0.00	0.0 - 4.5	1.00	1.0 - 1.0		

Ratio of mean epithelial reflectivity to stromal reflectivity

On ROC analysis, the area under the curve (AUC) was 0.970 (95% CI=0.92 to 0.99) with a z statistic of 26.34 ($p < 0.0001$). A score of >1.29 corresponded with the highest Youden index. The sensitivity at this threshold was 91.67 (95% CI: 80 to 97.7) and specificity of 98.75 (93.2 to 100). The positive likelihood ratio was 73.3 (95% CI=10.4 to 515.2), meaning a score of >1.29 was 73.3 times more likely in eyes with LSCD, than in eyes with non-LSCD. The negative likelihood ratio was 0.084 (95% CI=0.02 to 0.2), meaning a score of <1.29 was 8 times more likely in eyes with non-LSCD than in LSCD.

Table S13: Area under ROC curve for Ratio of Mean epithelial reflectivity to stromal reflectivity

**Area under the ROC curve (AUC)**	0.970
**Standard Error [a]**	0.0178
**95% Confidence interval [b]**	0.924 to 0.992
**z statistic**	26.346
**Significance level P (Area=0.5)**	<0.0001

[a] DeLong et al., 1988

[b] Binomial exact

Table S14: Youden index for Ratio of Mean epithelial reflectivity to stromal reflectivity

**Youden index J**	0.9042
**Associated criterion**	>1.290909091
**Sensitivity**	91.67
**Specificity**	98.75

Table S15: Criterion values and coordinates of the ROC curve

Criterion	Sensitivity	95% CI	Specificity	95% CI	+LR	95% CI	-LR	95% CI
≥0.557082371	100.00	92.6 - 100.0	0.00	0.0 - 4.5	1.00	1.0 - 1.0		
>0.833247789	100.00	92.6 - 100.0	31.25	21.3 - 42.6	1.45	1.3 - 1.7	0.00	

>0.841131972	97.92	88.9 - 99.9	31.25	21.3 - 42.6	1.42	1.2 - 1.7	0.067	0.009 - 0.5
>0.976744186	97.92	88.9 - 99.9	52.50	41.0 - 63.8	2.06	1.6 - 2.6	0.040	0.006 - 0.3
>1.011081016	95.83	85.7 - 99.5	52.50	41.0 - 63.8	2.02	1.6 - 2.6	0.079	0.02 - 0.3
>1.187503518	95.83	85.7 - 99.5	93.75	86.0 - 97.9	15.33	6.5 - 35.9	0.044	0.01 - 0.2
>1.191654142	93.75	82.8 - 98.7	93.75	86.0 - 97.9	15.00	6.4 - 35.2	0.067	0.02 - 0.2
>1.2130406	93.75	82.8 - 98.7	96.25	89.4 - 99.2	25.00	8.2 - 76.1	0.065	0.02 - 0.2
>1.263293808	91.67	80.0 - 97.7	96.25	89.4 - 99.2	24.44	8.0 - 74.4	0.087	0.03 - 0.2
**>1.290909091**	**91.67**	**80.0 - 97.7**	**98.75**	**93.2 - 100.0**	**73.33**	**10.4 - 515.2**	**0.084**	**0.03 - 0.2**
>1.479099678	62.50	47.4 - 76.0	98.75	93.2 - 100.0	50.00	7.0 - 355.0	0.38	0.3 - 0.5
>1.485714286	62.50	47.4 - 76.0	100.00	95.5 - 100.0			0.38	0.3 - 0.5
>4.464231425	0.00	0.0 - 7.4	100.00	95.5 - 100.0			1.00	1.0 - 1.0

Third parameter found to be significant – Mean superficial vascular density

On ROC analysis the area under the curve (AUC) was 0.914 (95% CI=0.85 to 0.95) with a z statistic of 17.03 ($p < 0.0001$). A score of >0.38 corresponded with the highest Youden index. The sensitivity at this threshold was 97.9 (95% CI: 88.9 to 99.9) and specificity of 73.75 (62.7 to 83). The positive likelihood ratio was 3.73 (95% CI=2.6 to 5.4), meaning a score of >0.38 was 3.73 times more likely in eyes with LSCD, than with in eyes with non-LSCD. While, the negative likelihood ratio was 0.028 (95% CI=0.02 to 0.2), meaning a score of <0.38 was 2.8 more likely in eyes with non-LSCD than in eyes with LSCD.

Table S16: Area under ROC curve for mean superficial vascular density

**Area under the ROC curve (AUC)**	0.914
**Standard Error [a]**	0.0243
**95% Confidence interval [b]**	0.852 to 0.956
**z statistic**	17.030
**Significance level P (Area=0.5)**	<0.0001

[a] DeLong et al., 1988
[b] Binomial exact

Table S17: Youden index for mean superficial vascular density

**Youden index J**	0.7167
**Associated criterion**	>0.38
**Sensitivity**	97.92
**Specificity**	73.75

Table S18: Criterion values and coordinates of the ROC curve

Criterion	Sensitivity	95% CI	Specificity	95% CI	+LR	95% CI	-LR	95% CI
≥0.28	100.00	92.6 - 100.0	0.00	0.0 - 4.5	1.00	1.0 - 1.0		
>0.35	100.00	92.6 - 100.0	38.75	28.1 - 50.3	1.63	1.4 - 1.9	0.00	
>0.36	97.92	88.9 - 99.9	53.75	42.2 - 65.0	2.12	1.7 - 2.7	0.039	0.006 - 0.3
**>0.38**	**97.92**	**88.9 - 99.9**	**73.75**	**62.7 - 83.0**	**3.73**	**2.6 - 5.4**	**0.028**	**0.004 - 0.2**
>0.39	83.33	69.8 - 92.5	82.50	72.4 - 90.1	4.76	2.9 - 7.8	0.20	0.1 - 0.4
>0.4	77.08	62.7 - 88.0	86.25	76.7 - 92.9	5.61	3.2 - 9.9	0.27	0.2 - 0.4
>0.41	68.75	53.7 - 81.3	90.00	81.2 - 95.6	6.88	3.5 - 13.6	0.35	0.2 - 0.5
>0.42	58.33	43.2 - 72.4	93.75	86.0 - 97.9	9.33	3.9 - 22.5	0.44	0.3 - 0.6
>0.43	43.75	29.5 - 58.8	96.25	89.4 - 99.2	11.67	3.7 - 37.1	0.58	0.5 - 0.8
>0.44	35.42	22.2 - 50.5	97.50	91.3 - 99.7	14.17	3.4 - 58.7	0.66	0.5 - 0.8
>0.45	18.75	8.9 - 32.6	97.50	91.3 - 99.7	7.50	1.7 - 33.3	0.83	0.7 - 1.0
>0.46	14.58	6.1 - 27.8	100.00	95.5 - 100.0			0.85	0.8 - 1.0
>0.48	0.00	0.0 - 7.4	100.00	95.5 - 100.0			1.00	1.0 - 1.0
